# Communications on Hand

**Published:** 1875-08

**Authors:** 


*Communications on Hand.*

On the Use of the Binder in Parturient Women, by Dr. P. H. Clark, Mason City, West Va.; Is Chorea a Sequence of Rheumatism? by Dr. J. C. Bishop, Middleport, O.; Ergot, by Dr. T. Curtis Smith, Middleport, O.; Opium in Pregnancy and Parturition, by Dr. Curtis Smith, Middleport, O.; Rötheln, by Dr. J. C. Bishop, Middleport, O.; Differential Symptoms of Epidemic Sore Throat and Diphtheria, by Dr. A. L. Knight, West Columbia, West Va.; Mastitis Treated with Phytolacca Decandra, by Dr. T. Curtis Smith, Middleport, O.; Plaster Paris in the Treatment of Fractures, by Dr. E. H. Trickle, Syracuse, O.; Duodenitis, by A. L. Knight, West Columbia, West Va.; The Old vs. The New, by A Medical Friend; Accumulation and Inspissation of Cerumen, by Dr. Swan M. Burnett, Knoxville, Tenn.; An Easily Affected Method of Artificial Respiration, by J. B. Mattison, Chester, N. J.; Cincho Quinine, by Dr. A. G. Smyth; What are the Causes of the Venous Circulation, author unknown; Abscess of Liver, by Dr. G. A. Baxter, Chattanooga, Tenn., as reported by Dr. Jas. B. Norris; Post Partum Hemorrhage, by Dr. F. K. Bailey, Knoxville, Tenn.; Case of Retroversion of the Uterus, by Dr. Patrick Booth, Walle county, N. C.

We take this opportunity of offering our most sincere and heartfelt thanks to our very able corps of contributors and co-laborers for the efforts they have made and the success they have attained in making the RECORD rank equal with any in our land. The above list of contributors would be an honor to any journal. With such noble hearts and highly cultivated intellects to aid our willing hands and grateful hearts, the RECORD, with all the troubles and difficulties usually attending the first five years of such publications, will live and grow, and be the pride and support of its readers. Will every reader do his duty? Will he see to it, that every worthy brother in his community has an opportunity to judge of its merits, to aid in its support, and enjoy its benefits and blessings? Go to work, friends, and determine to secure one new paying subscriber to commence with the January number, 1876.

